# Mitochondria Express α7 Nicotinic Acetylcholine Receptors to Regulate Ca^2+^ Accumulation and Cytochrome *c* Release: Study on Isolated Mitochondria

**DOI:** 10.1371/journal.pone.0031361

**Published:** 2012-02-16

**Authors:** Galyna Gergalova, Olena Lykhmus, Olena Kalashnyk, Lyudmyla Koval, Volodymyr Chernyshov, Elena Kryukova, Victor Tsetlin, Sergiy Komisarenko, Maryna Skok

**Affiliations:** 1 Department of Molecular Immunology, Palladin Institute of Biochemistry, Kyiv, Ukraine; 2 Department of Molecular Bases of Neurosignaling, Shemyakin-Ovchinnikov Institute of Bioorganic Chemistry, Russian Academy of Sciences, Moscow, Russia; City of Hope National Medical Center and Beckman Research Institute, United States of America

## Abstract

Nicotinic acetylcholine receptors (nAChRs) are ligand-gated ion channels that mediate synaptic transmission in the muscle and autonomic ganglia and regulate transmitter release in the brain. The nAChRs composed of α7 subunits are also expressed in non-excitable cells to regulate cell survival and proliferation. Up to now, functional α7 nAChRs were found exclusively on the cell plasma membrane. Here we show that they are expressed in mitochondria and regulate early pro-apoptotic events like cytochrome *c* release. The binding of α7-specific antibody with mouse liver mitochondria was revealed by electron microscopy. Outer membranes of mitochondria from the wild-type and β2−/− but not α7−/− mice bound α7 nAChR-specific antibody and toxins: FITC-labeled α-cobratoxin or Alexa 555-labeled α-bungarotoxin. α7 nAChR agonists (1 µM acetylcholine, 10 µM choline or 30 nM PNU-282987) impaired intramitochondrial Ca^2+^ accumulation and significantly decreased cytochrome *c* release stimulated with either 90 µM CaCl_2_ or 0.5 mM H_2_O_2_. α7-specific antagonist methyllicaconitine (50 nM) did not affect Ca^2+^ accumulation in mitochondria but attenuated the effects of agonists on cytochrome *c* release. Inhibitor of voltage-dependent anion channel (VDAC) 4,4′-diisothio-cyano-2,2′-stilbene disulfonic acid (0.5 µM) decreased cytochrome *c* release stimulated with apoptogens similarly to α7 nAChR agonists, and VDAC was co-captured with the α7 nAChR from mitochondria outer membrane preparation in both direct and reverse sandwich ELISA. It is concluded that α7 nAChRs are expressed in mitochondria outer membrane to regulate the VDAC-mediated Ca^2+^ transport and mitochondrial permeability transition.

## Introduction

Nicotinic acetylcholine receptors (nAChRs) are pentameric ligand-gated ion channels that were initially explored in muscle and autonomic ganglia and shown to mediate fast synaptic transmission [1]. In the brain, they regulate glutamate-, GABA- and dopamine-mediated transmission and are involved in the establishment of nicotine dependence [2]. Studies of the last decade documented the presence of nAChRs and nAChR-like receptors in many non-excitable cells of mammals, as well as in invertebrates, plants and even bacteria, where their functions are related to the general vital properties of living cells like proliferation, survival, adhesion and motility [3]–[5]. It is becoming increasingly clear that nAChRs have appeared in evolution long before the development of the nervous system and that they are multifunctional receptors employing different kinds of signaling in the cells of various origin.

Structurally, the nAChRs are composed of combinations of ten alpha (α1 to α10) and four beta (β1 to β4) subunits, muscle-type receptors contain also γ, δ or ε subunits. They can be assembled as heteropentamers (eg. (α1)_2_β1γδ (α3)_2_(β4)_3_, (α3)_2_α5(β4)_2_, (α4)_2_(β2)_3_, etc.) or as homopentamers (eg. (α7)_5_) and, correspondingly, differ in their cation selectivity, kinetics of the ion channel opening and desensitization [1].

The homopentameric nAChRs composed of α7 subunits (α7 nAChRs) are of special interest, because they belong to the most ancient branch of this receptor's family and were shown to be expressed in both neurons and non-excitable cells to mediate pro-proliferative, survival and anti-inflammatory signaling [6]–[9]. Previously we found that the absence of these receptors in α7−/− mice resulted in poorer survival of B lymphocyte precursors within the bone marrow [7]. Activation of nAChRs stimulated the growth of cancer cells and suppressed apoptosis [10], and the nAChR agonist nicotine could abolish the chemotherapy-induced apoptosis [11]. However, up to now, the pro-survival signaling was attributed exclusively to α7 nAChRs exposed on the cell plasma membrane. We posed a question: whether functional α7 nAChRs can be found in intracellular organelles, in particularly, in mitochondria, which are involved in inducing intracellular apoptotic pathway? Here we show that α7 nAChRs are expressed in the outer mitochondria membrane to regulate Ca^2+^ accumulation and cytochrome *c* release stimulated with apoptogens like high Ca^2+^ dose or H_2_O_2_.

## Results

### The presence of α7 nAChRs in mitochondria

**Figure 1:**
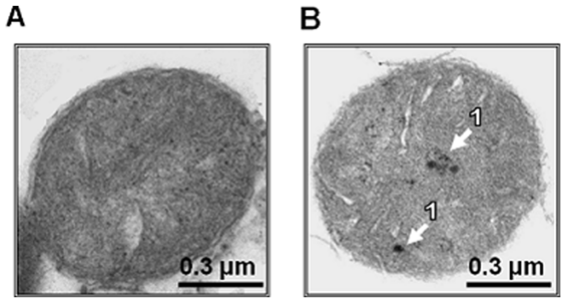
Electron microscopy images of mouse liver mitochondria. Isolated mitochondria were stained with rabbit α7(1–208)-specific antibody followed by 10 nm colloidal gold-conjugated anti-rabbit IgG. **A** – secondary antibody only (control), **B** – α7(1–208)-specific antibody plus secondary antibody; arrows (1) indicate the sites of positive staining.

Mitochondria isolated from the liver of C57Bl/6 mice were treated with the antibody against the whole extracellular domain (1–208) of α7 nAChR subunit, followed by the 10 nm colloidal gold conjugated secondary antibody, and examined by electron microscopy. As shown in Fig. 1, the binding of α7(1–208)-specific antibody was detected on mitochondria bodies. However, positive staining was quite rare, probably, due to the mode of sample processing (cutting) for electron microscopy.

**Figure 2:**
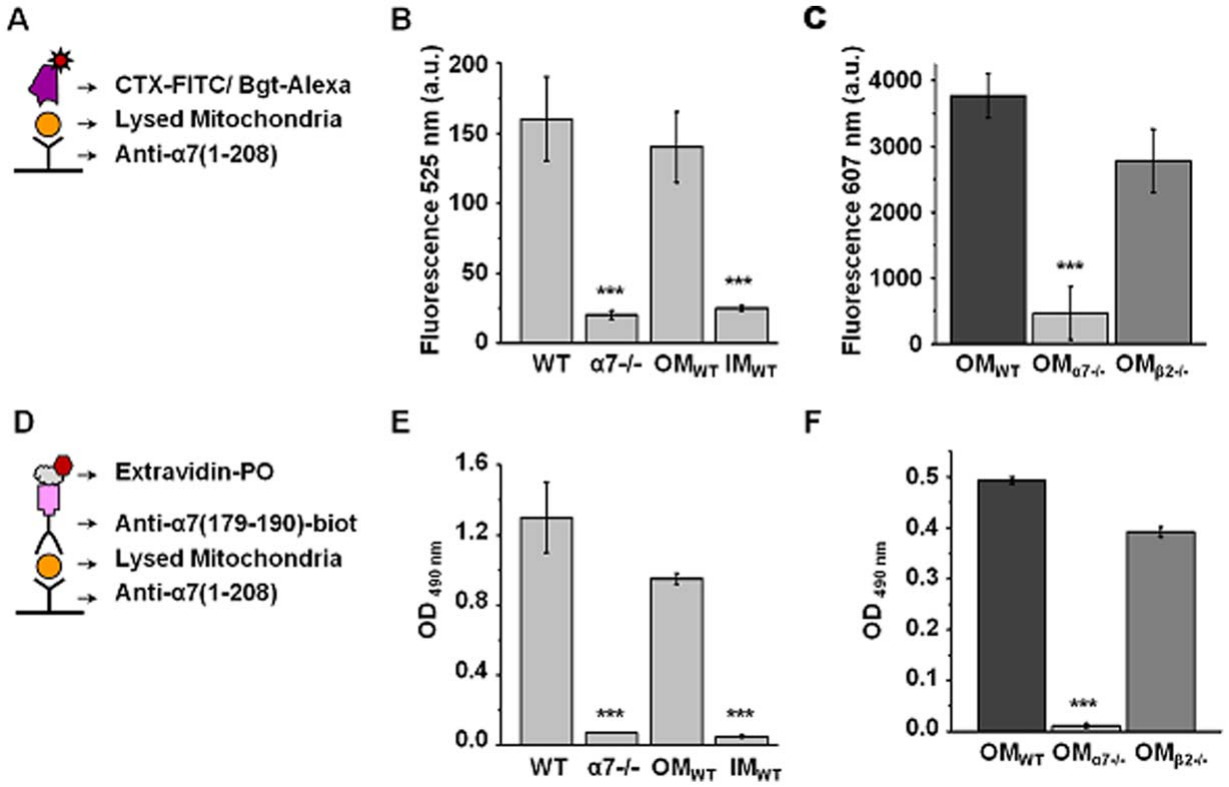
Identification of α7 nAChRs in isolated mitochondria by sandwich ELISA. **A** and **D** – schemes of assays used; CTX-FITC – FITC-labeled α-cobratoxin, Bgt-Alexa – Alexa Fluor 555-labeled α-bungarotoxin, PO – horseradish peroxidase. **B** and **E** – the results of the CTX-FITC-developed (**B**, n = 6) or antibody-developed (**E**, n = 4) sandwich assays with the lysates of non-fractionated mitochondria from the wild-type (WT) or α7−/− mice and the outer (OM) and inner (IM) membranes of the wild-type mitochondria. *** – *P<0.0005* compared to the data of the wild-type mitochondria and their outer membranes. **C** and **F** – the results of Bgt-Alexa-developed (**C**, n = 8) or antibody-developed (**F**, n = 5) sandwich assays with the outer membranes of mitochondria from the wild-type, α7−/− or β2−/− mice; non-specific binding detected in the presence of 200-fold molar excess of non-labeled α-cobratoxin (**C**) is subtracted. *** – *P<0.0005* compared to the data of the outer membranes of the wild-type mitochondria.

As the next step, we studied the presence of nAChRs in detergent lysates of mitochondria isolated from the liver of either wild-type or α7−/− mice. For this purpose, two types of sandwich assays were developed (Fig. 2, A, D). The nAChR contained within the mitochondria preparation was captured with the antibody against á7(1–208) and was further revealed with either fluorescein isothiocyanate-labeled α-cobratoxin (CTX-FITC) or α7(179–190)-specific antibody. CTX is a long-chain α-neurotoxin from *Naja kaouthia* cobra venom; a specific ligand for the muscle-type, á7 and á9(á10) nAChRs of mammals [12]. Antibody against the extracellular epitope (179–190) of α7 nAChR subunit was generated by us previously [13] and was proven to be α7-specific in numerous experimental systems and assays including ELISA, Western blot and flow cytometry [7], [14]. As shown in Fig. 2, B and E, the binding of both toxin and antibody was observed with the mitochondria of the wild-type but not α7−/− mice in two independent assays. When the wild-type mitochondria were fractionated into the outer (OM) and inner (IM) membranes, the binding of both CTX-FITC and á7(179–190)-specific antibody was found with the OM, but not IM preparation (that explained the rare positive staining of cut mitochondria in electron microscopy, Fig. 1). We further compared the OM preparations of the wild-type, α7−/− and β2−/− mice in the antibody- and toxin-based sandwich assays; in this case we used Alexa Fluor 555-labeled α-bungarotoxin previously shown to bind specifically α7 nAhCRs in both α7-transfected model cells and in dorsal-root ganglia naturally expressing this nAChR subtype [15]. As shown in Fig. 2, C and F, positive signal was found in the preparations of the wild-type and β2−/−, but not α7−/− mitochondria. This data clearly indicated that α7 nAChR was present in the mitochondria outer membrane.

### Regulation of Ca^2+^ accumulation in mitochondria by α7 nAChR ligands

**Figure 3:**
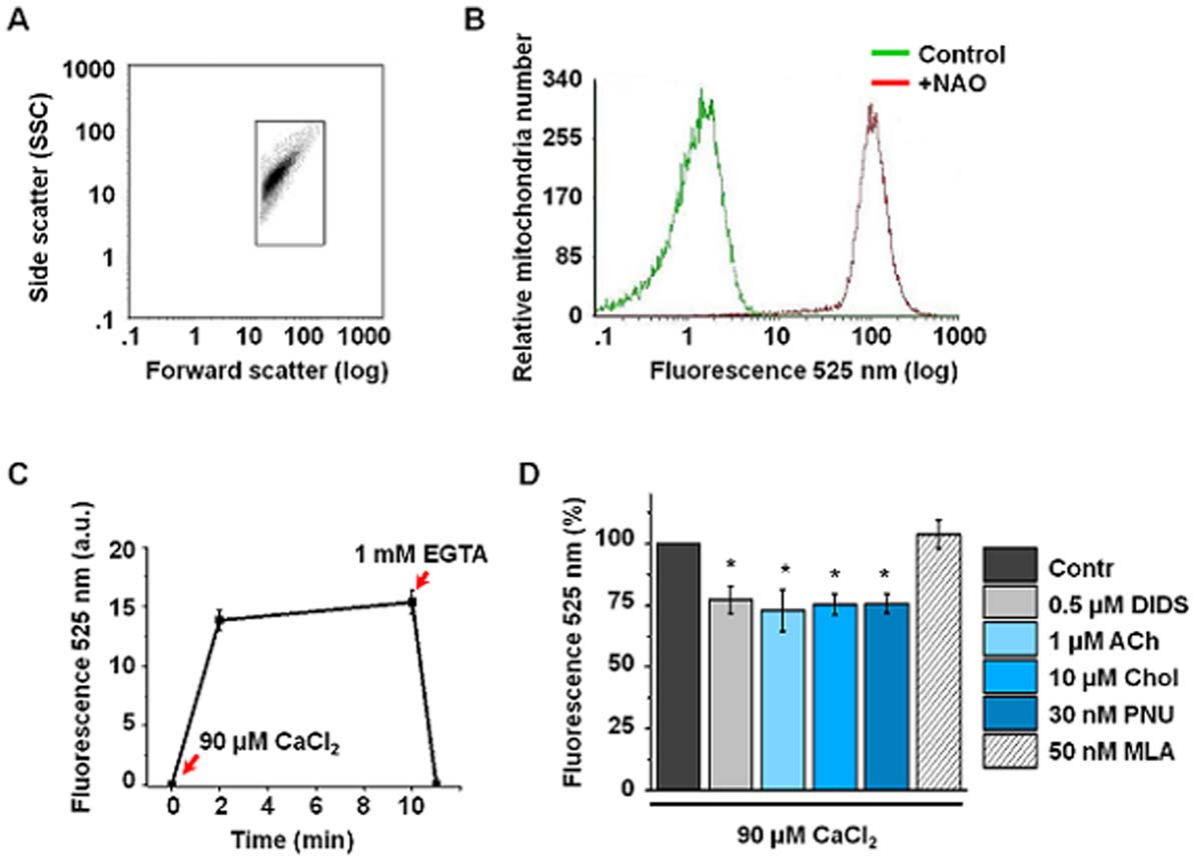
The effects of α7-specific ligands and DIDS on Ca^2+^ accumulation in mitochondria studied by flow cytometry. **A** – purified liver mitochondria gated by size (Forward scatter) and granularity (Side scatter) in flow cytometry. **B** – binding of 0.1 µM acridine orange 10-nonyl bromide (NAO) to the gated mitochondria population; Control – the non-stained mitochondria. **C** – Ca^2+^ accumulation in mitochondria loaded with Fluo 3-AM. **D** – Ca^2+^ accumulated in mitochondria during 2 min after 1 min pretreatment with DIDS, acetylcholine, choline, PNU-282987 or MLA; data are shown as normalized mean fluorescence values of 5 independent experiments for each ligand; * – *P<0.05* compared to the fluorescence in the absence of α7 nAChR agonists or DIDS.

To reveal possible functions of α7 nAChRs in mitochondria, we took into account that this nAChR subtype is highly permeable to Ca^2+^ [1], whereas mitochondria are well-known intracellular Ca^2+^ depots. To find out whether mitochondrialα7 nAChRs are involved in Ca^2+^ transport we studied intramitochondrial Ca^2+^ accumulation in the presence or absence of α7-specific ligands. To exclude any contamination with the whole cells or plasma membranes, the isolated mitochondria were allocated by flow cytometry according to their size and granularity (Fig. 3, A). 95% of particles within the gated population incorporated mitochondria-specific fluorescent dye acridine orange 10-nonyl bromide [16] indicating that it contained pure mitochondria (Fig. 3, B). Experiments were performed in live mitochondria maintaining their membrane potential monitored with the potential-sensitive fluorescent dye tetramethyl rhodamine methyl esther [17]. Addition of 90 µM CaCl_2_ to the mitochondria loaded with Ca^2+^-sensitive fluorescent dye Fluo 3-AM evoked the fluorescent signal within the first two minutes; the signal was maintained during at least 10 min more and was completely abolished by the addition of 1 mM EGTA (Fig. 3, C) or 0.1 µM carbonyl cyanide-3-chlorophenylhydrazone (CCCP, data not shown). When CaCl_2_ application was preceded with that of α7 nAChR agonists choline, acetylcholine or PNU-282987, but not a specific competitive antagonist methyllicaconitine (MLA), Ca^2+^ accumulation was inhibited by about 20% (Fig. 3, D). Similar effect was exerted by the VDAC inhibitor 4,4′-diisothiocyano-2,2′-stilbene disulfonic acid (DIDS). VDAC is also located in mitochondria outer membrane and facilitates Ca^2+^ entry from the cytosol into the intermembrane mitochondria space [18]. Similar effects of DIDS and α7 nAChR agonists on Ca^2+^ accumulation in mitochondria pushed us to search a further physical and functional connection between VDAC and α7 nAChR.

### Connection of α7 nAChR to VDAC in the outer mitochondria membrane

**Figure 4:**
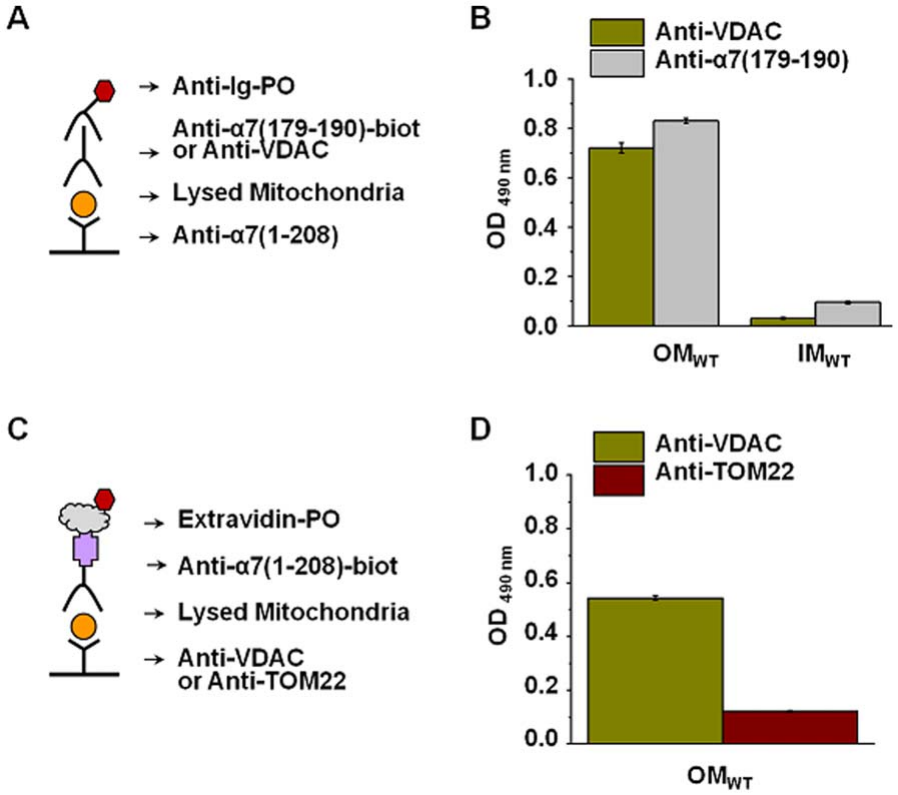
Connection of α7 nAChR and VDAC studied by sandwich assays. The schemes (**A, C**) and results of direct (**B**, n = 3) and reverse (**D**, n = 3) sandwich assays demonstrating the connection of α7 nAChR and VDAC in the outer membranes (OM) of the wild-type (WT) mitochondria. Anti-TOM22 – antibody against mitochondrial outer membrane translocase.

To elucidate if there is an interaction between α7 nAChR and VDAC in the outer mitochondria membrane we developed another set of sandwich immunoenzyme assays presented in Fig. 4 A, C. These assays are analogues of immunoprecipitation where the complex of two interacting proteins is captured from the mixture with the antibody against one component and is revealed in Western blot with the antibody against another component. The advantage of sandwich assay is that both steps are performed in the same media (immunoplate) and that the second antibody binding is evaluated photometrically as in conventional ELISA. Previously, we used such an approach to reveal the interaction of α7 nAChR with CD40 and of α4β2 nAChRs with IgM in B lymphocytes [19]. In the first assay, the α7 nAChR was captured from the OM preparation with anti-α7(1–208) and was revealed with either anti-α7(179–190) or anti-VDAC (Fig. 4, A). As shown in Fig. 4, B, the OM preparation captured with anti-α7(1–208) was revealed with both anti-VDAC and anti-α7(179–190). In the “reverse” assay (Fig. 4, C), the complex was captured with either anti-VDAC or the antibody against mitochondria outer membrane translocase (anti-TOM22) and was revealed with anti-α7(1–208). In this case, the nAChR-specific antibody recognized the complex captured with anti-VDAC and not with anti-TOM22 (Fig. 4, D). These data clearly demonstrated that α7 nAChR interacts with the VDAC in the outer mitochondria membrane.

### Regulation of cytochrome *c* release from mitochondria by α7 nAChR ligands

**Figure 5:**
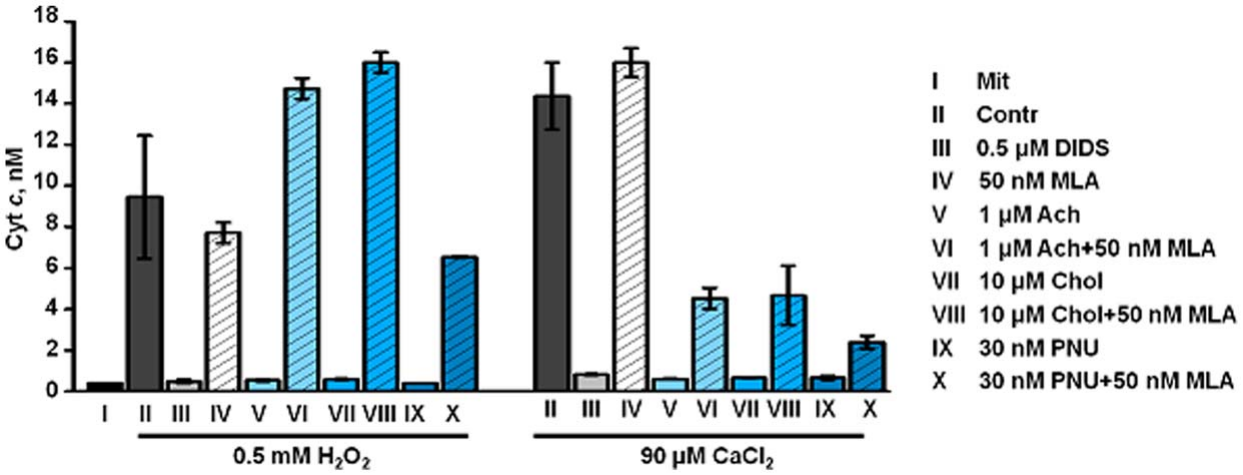
Cytochrome *c* release into mitochondria supernatants. Cyt *c* released from mitochondria in 2 min after addition of either 0.5 mM H_2_O_2_ or 90 µM CaCl_2_ in the presence or absence of DIDS or α7 nAChR ligands. Each column corresponds to M±SE of three independent measurements. Mit – mitochondria without apoptogens; Contr – mitochondria treated with H_2_O_2_ or CaCl_2_ only.

VDAC is a key element in mitochondria permeability transition pore (MPTP) formation accompanied with cytochrome *c* (cyt *c*) release [20] which is the initial step of mitochondria-driven apoptosis. To test if mitochondrial α7 nAChRs are involved in apoptosis-related processes, we studied the effects of á7 nAChR ligands on the cyt *c* release from purified mitochondria stimulated with either high dose of CaCl_2_ or H_2_O_2_. Preliminary data demonstrated that cytochrome *c* release stimulated with 0.5 mM H_2_O_2_ was inhibited with α7 nAChR agonists and DIDS [21]. Here we show that 90 µM CaCl_2_ stimulated extensive cyt *c* release from live mitochondria similarly to 0.5 mM H_2_O_2_; both were significantly inhibited with 0.5 µM DIDS or á7 nAChR agonists (choline, acetylcholine or PNU-282987, Fig. 5). MLA did not affect cyt *c* release itself but prevented inhibitory effects of all agonists. This data clearly indicated that á7 nAChRs were involved in regulating MPTP formation, and their activation with agonists produced an effect similar to inhibition of VDAC by DIDS.

## Discussion

The presence of nicotinic receptors in mitochondria has initially been discussed in connection with the neuroprotective role of nicotine. Then it was shown that nicotine affected mitochondria respiratory chain independently on nAChRs [22]. However, in other studies, the decrease of mitochondria membrane potential caused by ethanol was prevented with specific α7 nAChR agonist 2,4-dimethoxibenziliden anabasein and this effect was blocked with MLA [23]. In the latter work, α7 nAChR agonists also attenuated cytochrome *c* release stimulated by ethanol in rat hippocampal neuronal cultures that is in good agreement with our data. In our earlier published experiments, 1 µM nicotine prevented Ca^2+^ accumulation in isolated mitochondria similarly to 1 µM choline, and mitochondria from mice injected with α7(1–208)-specific antibody possessed lower membrane potential than those from mice injected with non-specific IgG, suggesting the involvement of α7 nAChRs [24]. The binding of α7-specific antibody with mitochondria of rat hippocampus was demonstrated by electron microscopy; however, this was not confirmed by Western blot analysis [25] and, therefore, did not allow the authors to state the expression of α7 nAChRs in mitochondria.

Our data clearly demonstrate that nAChRs of α7 subtype are present in the outer membranes of mitochondria isolated from the mouse liver. This was shown by electron microscopy with α7(1–208)-specific antibody and by sandwich ELISA with α7(179–190)-specific antibody. Since the specificity of many antibodies against nAChRs was put in doubt [26]–[27], we confirmed our results by independent binding of either α-cobratoxin or α-bungarotoxin fluorescent derivatives and used mitochondria from the wild-type, α7−/− or β2−/− mice to prove the specificity of binding (Fig. 2 B, C). Mouse liver is a recognized source for mitochondria isolation [28]; it is yet to be established if mitochondria from other tissues and species contain similar or different nAChR quantity and/or subtype.

To reveal the functions of α7 nAChRs in mitochondria, we took into account that nAChRs expressed in non-excitable cells trigger intracellular signaling and affect the activity of adjacent receptors by either ion-dependent or independent mechanism [29]–[31]. Previously we found that α7 nAChR expressed in mouse B lymphocytes was coupled with CD40 and regulated CD40-mediated B lymphocyte activation [19]. Similar approach applied to mitochondria indicates that mitochondrial α7 nAChR is coupled to VDAC that may underlie similar effects of α7 agonists and DIDS on Ca^2+^ accumulation and cyt *c* release (Fig. 3, D and 5).

VDAC, positioned on the interface between mitochondria and the cytosol, is responsible for the fluxes of various metabolites across mitochondria outer membrane [32]–[33]. It is suggested to increase the local Ca^2+^ concentration in the intermembrane mitochondria space thus facilitating the Ca^2+^uniporter activity [34]. It has been also recognized as a key protein in MPTP formation and induction of mitochondria-mediated apoptosis [35]. VDAC is easily converted from anion-selective to cation-permeable state by environmental conditions [36] and is oligomerized to be involved in MPTP [18]. Similar effects of α7 nAChR agonists and of DIDS, which prevents VDAC's oligomerization [18], allow suggesting that mitochondrial α7 nAChR signaling affects the neighboring VDAC thus preventing its involvement in MPTP. Interestingly, the doses of choline (10 µM) and acetylcholine (1 µM) affecting both Ca^2+^ accumulation and cyt *c* release were much lower than those reported to open the α7 nAChR ion channel (EC_50_ 1.6 mM for choline and 79–316 µM for acetylcholine); that of synthetic agonist PNU-282987 (30 nM) was closer to but still less than its reported functional potency (128 nM) [37]. Since the effects of all tested agonists on cyt *c* release were inhibited with the competitive inhibitor MLA, it may be suggested that mitochondrial α7 nAChRs are much more sensitive to natural agonists than those expressed in the plasma membrane, possibly, due to specific lipid surrounding of mitochondria outer membrane [38]. The lipids were shown to influence the ability of the nicotinic acetylcholine receptor to gating in response to neurotransmitter binding [39]. In addition, the exact subunit composition of α7-containing mitochondrial nAChRs is still to be elucidated, since heteromeric α7β2 nAChRs were shown to possess different pharmacological sensitivity compared to α7 homopentamers [40].

Recognizing the α7 nAChR presence in mitochondria poses a question about its natural ligand(s) and physiological significance. Choline is actively transported into the cell from extracellular space and is present in the cytosol at 30 to 50 µM [41]. According to our data, it is sufficient to activate mitochondrial α7 nAChRs and to keep mitochondria “resistant” to apoptogenic agents. However, the half-life time of intracellular choline is very short [42], therefore, the mitochondria protection depends on the balance of its intake and degradation. Recent evidence demonstrates the increased susceptibility of mitochondria to calcium-induced permeability transition upon choline deficiency [43] that supports our idea. In contrast, intracellular release of choline upon ischemia [42] obviously has the protective anti-apoptotic effect. In addition, according to proteomic studies, mitochondria contain choline acetyltransferase [44] and, therefore, are able to locally synthesize acetylcholine.

In summary, our results demonstrate that á7 nAChRs are expressed on mitochondria outer membrane and regulate Ca^2+^ accumulation and cyt *c* release, the initial step of apoptosis induction. This means that, in addition to established anti-apoptotic signaling pathways mediated by plasma membrane α7 nAChRs [8]–[9], there is an endogenous, previously unrecognized cholinergic mechanism to control mitochondria functions and their apoptotic susceptibility. Probably, it belongs to the most ancient survival mechanisms inherited by mitochondria from their hypothetic prokaryotic ancestor [45]. This finding offers a novel view on the mitochondria protection in apoptosis and opens the way for its pharmacological regulation.

## Materials and Methods

### Ethics Statement

We used age-matched male wild-type and mutant (lacking either α7 or β2 nicotinic receptor subunit [46]–[47]) mice with common C57BL/6J background. The mice were kept in the animal facilities of Pasteur Institute, Paris and Palladin Institute of Biochemistry, Kyiv. They were housed in a quiet, temperature-controlled room (22–23°C) and were provided with water and dry food pellets *ad libitum*. Before removing the liver mice were sacrificed by cervical dislocation. All procedures conformed to the guidelines of the Centre National de la Recherche Scientifique or IACUC of Palladin Institute. Before starting the experiments, the protocols were approved by the Animal Care and Use Committee of Palladin Institute of Biochemistry (Protocol 1/7-421).

### Reagents

All reagents were of chemical grade and were purchased from Sigma-Aldrich unless specially indicated. Alexa Fluor 555 α-bungarotoxin was purchased from Invitrogen (USA). CTX-FITC and antibodies against α7(1–208) or α7(179–190) were obtained by us previously [13], [48]–[49].

### Mitochondria purification and fractionation

Mitochondria isolation from the mouse liver and fractionation into inner and outer membranes was performed by differential ultracentrifugation according to standard procedure described [50]. The separation medium contained 10 mM HEPES, 1 mM EGTA and 250 mM sucrose, pH 7.4, 4°C. For the membrane preparation, the isolated mitochondria were resuspended in 10 ml 10 mM KH_2_PO_4_, left on ice for 5 min and spun at 8000 g for 10 min. The pellet was resuspended in 10 ml 125 mM KCl, 10 mM Tris-MOPS (pH 7.4, 4°C) and centrifuged again. The supernatant was withdrawn, while the pellet was resuspended in the mixture of 10 mM KH_2_PO_4_ (10 ml) and 1.8 M sucrose, 2 mM ATP, 2 mM MgSO_4_ (3.5 ml). The sample was sonicated at 4 W for 20 min, laid on the top of 15 ml 1.18 M sucrose and spun for 2 h at 90 000 g. The inner membranes were collected as the brown pellet in the tube bottom, while the outer membranes were found precipitated in the interphase. To prepare detergent lysates, the mitochondria suspension or membrane fractions were freezed at −70°C, thawed and treated with the lysing buffer (0.01 M Tris –HCl, pH 8.0; 0.14 M NaCl; 0.025% NaN_3_; 1% Tween 20) and protease inhibitors cocktail for 2 h on ice upon intensive stirring. The resulting lysate was cleared by centrifugation (20 min at 20 000 g) and dialysed against PBS containing 0.025% NaN_3_ and protease inhibitors. The protein concentration was established by Bradford assay.

### Electron microscopy

The purified mitochondria were treated in suspension with α7(1–208)-specific rabbit antibody (0.05 mg/ml) for 30 min at RT, washed by centrifugation and subsequently incubated with 10 nm colloidal gold-labeled goat anti-rabbit immunoglobulins for additional 30 min. Control preparation was treated with the second antibody only. The washed mitochondria were pelleted and fixed with 2.5% glutaraldehyde in 0.1 M sodium cacodylate buffer pH 7.4 during 3 h at RT. The samples were additionally fixed with 1% OsO_4_ in 0.05 M cacodylate buffer during 2 h and were dehydrated in 30%, 50%, 70%, 90% and 100% acetone subsequently. The obtained preparations were polymerized in epoxy resin Apon-Araldyt (AGAR, UK). Ultrathin lesions (70–100 nm) were prepared with Ultratome LKB-V (LKB, Sweden) and were stained with 1% uranyl acetate (60 min) and the lead dye (2 min). The preparations were analyzed under electron microscope H-600 (Hitachi, Japan) with 20,000 amplification.

### Flow cytometry studies

The purified mitochondria (200 µg of protein per ml in the standard sample) were resuspended in the incubation medium containing 10 mM HEPES, 125 mM KCl, 25 mM NaCl, pH 7.4. For Ca^2+^-related studies the incubation medium was supplemented with 5 mM sodium succinate and 0.1 mM Pi(K), pH 7.4. The purity of gated mitochondria in flow cytometry was assessed using 0.1 µM acridine orange 10-nonyl bromide (NAO) added immediately before flow cytometry examination. For Ca^2+^ uptake studies, mitochondria were pre-incubated with 1 µM Fluo 3-AM for 30 min in the dark. CaCl_2_, DIDS and α7 nAChR ligands were added as described in the Fig. 3 and its legend. The studies were performed with the COULTER EPICS-XL™ fluorescent flow cytometer (Beckman Coulter, USA) at room temperature.

### Sandwich assays

The 96-well plates (Nunc MaxiSorp, Denmark) were coated with rabbit α7(1–208)-specific antibody and were subsequently blocked with 1% BSA/PBS. The detergent lysates of mitochondria or their membranes were applied into the coated wells, 600 µg/ml (for the whole lysate) or 100 µg/ml (for membranes). After 2 h of incubation at 37°C the plates were washed with water. In ELISA version, the bound antigen was revealed with biotinylated rabbit α7(179–190)-specific antibody or VDAC-specific antibody (Santa Cruz Biotechnology, USA) applied for additional 2 h and followed by Extravidin-peroxidase conjugate and *o*-phenylenediamine-containing substrate solution. The absorbance at 490 nm was read by the StatFax-2100 Microplate reader (Awareness Technology, USA). In the combined antibody-toxin version, the bound antigen was detected with either CTX-FITC (10–20 µg/ml) or Alexa Fluor 555-labeled α-bungarotoxin in 50 nM final concentration applied for 1 h (CTX) or overnight (α-bungarotoxin) at RT and washed out with PBS. The fluorescence of dry plates was then read with FLx800 Multi-detection microplate reader (BioTek, USA) or fluorimeter FFM-01 (Kortek, Russia) using excitation/emission wavelength of 485/530 nm or 546/607, respectively.

### Cyt *c* release studies

The purified mitochondria (120 µg of protein per ml) were incubated with either 90 µM CaCl_2_ or 0.5 mM H_2_O_2_ in the presence or absence of DIDS or nAChR ligands for 2 min at room temperature and were immediately pelleted by centrifugation (10 min., 7000 g) at 4°C. The supernatants were collected and tested by sandwich assay. The plates were coated with ammonium sulfate-precipitated fraction of bovine cyt *c*-specific rabbit antiserum and blocked with 1% BSA. Mitochondria supernatants were applied at optimal dilutions established in preliminary experiments. Calibration curve was built using bovine cyt *c*. The bound cyt *c* was revealed with biotinylated immunoglobulins from cyt *c*-specific rabbit serum followed by Extravidin-peroxidase conjugate and *o*-phenylendiamin-containing substrate solution. This assay became possible because of multiple epitopes recognized on cyt *c* by polyclonal antibodies and due to evolutional conservatism of cyt *c* molecule, so that antibodies raised against bovine cyt *c* could recognize its mouse analogue.

### Statistical analysis

Each experiment was reproduced independently minimum three times with 3 to 6 repeats for each point. Statistical analysis was performed according to Student's test using OriginPro 8.6 software. The data are presented as mean and SEM. The difference was considered significant at P<0.05 (*).
